# Effect of Diabetes Self-Efficacy on Coping Strategy: Self-Stigma’s Mediating Effect

**DOI:** 10.3390/healthcare13091066

**Published:** 2025-05-05

**Authors:** Hyunjin Lee, Seyeon Park, Kawoun Seo

**Affiliations:** 1 College of Nursing, Eulji University, Uijeongbu-si 11749, Republic of Korea; hjlee@eulji.ac.kr; 2 College of Nursing, Chungnam National University, Daejeon 35015, Republic of Korea; 3 Department of Nursing, Joongbu University, Chungnam 32713, Republic of Korea

**Keywords:** coping strategy, diabetes mellitus, mediation analysis, self-efficacy, social stigma

## Abstract

**Objectives:** This descriptive study aimed to explore self-stigma’s mediating effect on the association between self-efficacy and coping strategy among Korean patients with type 2 diabetes. **Methods:** This study included 189 participants with type 2 diabetes diagnosed by an endocrinologist. Data were collected from 1 November to 28 December 2023, using a self-report questionnaire focusing on self-efficacy, coping strategy, and self-stigma. The collected data were analyzed using descriptive statistics, independent sample *t*-test, one-way analysis of variance, Pearson’s correlation coefficient, and multiple regression analysis using SPSS; the mediating effect was examined using SPSS PROCESS Macro. **Results:** The mean self-efficacy, coping strategy, and self-stigma scores were 6.29 (±10.80), 2.84 (±0.54), and 2.72 (±0.82), respectively. Self-efficacy was negatively correlated with coping strategy (r = −0.52, *p* < 0.001) and self-stigma (r = −0.45, *p* < 0.001). Coping strategy was positively correlated with self-stigma (r = 0.78, *p* < 0.001). Further, self-stigma partially mediated the relationship between self-efficacy and coping strategy, accounting for 64.0% of the variance. **Conclusions:** These results suggest the importance of tailoring self-stigma reduction strategies to enable patients with diabetes to develop positive coping strategies. Moreover, incremental and tailored programs for reducing self-stigma must be developed.

## 1. Introduction

Diabetes is a chronic condition impacting people worldwide. The International Diabetes Federation estimates that, by 2045, 1 in 8 adults (approximately 783 million individuals) will have diabetes [1]. In South Korea, as of 2022, one in seven adults aged 30 years or older has diabetes, 90% of whom have type 2 diabetes [1,2]. Diabetes—a complex condition which poses significant challenges to its management—can precipitate severe complications, including kidney disease, retinopathy, and cardiovascular disorders [3]. Poor blood sugar control not only heightens the risk of these complications but also contributes to increased depression, stress, and diminished quality of life among patients [3,4,5]. Consequently, identifying effective management strategies to regulate blood sugar levels and prevent long-term complications is crucial to improving outcomes for patients with diabetes.

Self-efficacy—an individual’s belief in their ability to successfully perform specific behaviors—is a critical factor in promoting behavioral change and achieving positive outcomes in addressing health problems [6]. Patients with a low self-efficacy frequently feel inadequate and are more likely to avoid or quickly abandon necessary behaviors [6,7,8]. By contrast, patients with a high self-efficacy consistently engage in disease management behaviors, which positively impact blood sugar control, support healthy lifestyle habits, and help prevent complications [8]. Thus, recognizing and enhancing the self-efficacy of patients with diabetes is essential for successful self-care.

Managing diabetes requires ongoing and complex self-care, and patients typically face challenging situations and stress during this process [9]. Appropriate coping strategies are crucial for navigating these challenges, adapting to stressors, and sustaining self-management [10,11,12]. Coping strategies are typically categorized into problem- and emotion-focused coping [13]. Specifically, emotion-focused coping strategies focus on managing the emotional distress associated with stressors but may occasionally result in inadequate self-management, thus complicating disease control and exacerbating psychosocial issues [12]. By contrast, problem-focused coping strategies, which involve actively addressing stressors, support positive health outcomes, such as improved blood sugar control [12]. Specifically, problem-focused coping entails efforts to directly resolve stressors, whereas emotion-focused coping involves efforts to alleviate the emotional impact of stress [11,13]. These coping styles are shaped by an individual’s psychological traits and beliefs, and self-efficacy plays a particularly significant role in determining the choice of coping strategy [7,11,14,15]. Thus, a deeper understanding of the coping strategies employed by patients with diabetes is vital for developing effective management approaches.

Furthermore, individuals with type 2 diabetes may experience self-stigma because of internalizing negative social stereotypes or biases regarding their condition [16]. Self-stigma creates psychological barriers that foster a negative perception of illness and, consequently, reduce self-efficacy and impede effective coping strategies [16,17]. Studies have demonstrated that patients with high self-stigma levels are less motivated to self-manage and are more likely to adopt ineffective coping strategies [16,17]. Consequently, self-efficacy is considered to play an important role in shaping how patients manage their disease and influencing self-stigma. That is, high self-efficacy is expected to mitigate self-stigma, which is a negative bias toward oneself related to diabetes; further, reduced self-stigma is expected to improve coping strategies for diabetes. However, research on self-stigma among patients with diabetes is limited. In particular, research on self-stigma’s role in the relationship between self-efficacy and coping strategies is extremely limited.

This study aims to examine the levels of self-efficacy, coping strategies, and self-stigma among patients with type 2 diabetes and investigate whether self-stigma mediates the relationship between self-efficacy and coping strategies. Thereby, this study seeks to provide a deeper understanding of self-stigma’s psychological impact on disease management and offer foundational data for developing effective nursing interventions.

## 2. Materials and Methods

### 2.1. Study Design

This descriptive study explored self-stigma’s mediating effect on the relationship between self-efficacy and coping strategies among patients with type 2 diabetes.

### 2.2. Participants

This study’s participants were adults diagnosed with type 2 diabetes by a doctor. The specific inclusion criteria were as follows: (1) adults aged 19 years or older who were diagnosed with type 2 diabetes by a doctor; (2) those with physical and cognitive abilities that enabled self-care for diabetes; (3) those who could understand and respond to the questionnaire; and (4) those who voluntarily agreed to participate in the survey and completed it.

The number of participants required for the multiple regression analysis was calculated using the G*power 3.1 program. When the effect size was 0.15, the significance level (α) was 0.05, the power was 0.90, and 13 general characteristics were entered; self-efficacy and self-stigma were independent variables, and coping strategy was the dependent variable. The required number of participants was 162. However, to ensure the fidelity of the survey results, 200 questionnaires were collected, 11 of which were insincerely filled out to and thus excluded; the final number of participants was 189.

### 2.3. Measures

#### 2.3.1. Independent Variable: Self-Efficacy

Self-efficacy was measured using the Korean version of the diabetes self-efficacy tool developed by the Stanford Patient Education Research Center [18], modified and supplemented by Chang et al. [19]. This tool comprised 8 items, each measured on a 10-point Likert scale. A higher score indicated a higher self-efficacy level in diabetes self-care. Negative items were reverse-coded. In Chang et al.’s study [19], the tool’s reliability coefficient was Cronbach’s α = 0.89, and in this study, its reliability coefficient was Cronbach’s α = 0.87.

#### 2.3.2. Dependent Variable: Coping Strategy

Coping strategy was measured using the Korean version of the diabetes coping strategy tool developed by Welch [20]—adapted to the Korean context by Byun [21]. This tool comprised 21 items, each measured on a 5-point Likert scale. Items 1, 4, 9, 12, and 20 were reverse-coded. A higher score indicated a lower coping strategy level. However, in the mediation analysis, a reverse-transformed score was used such that a higher score indicated a higher coping strategy level. In Byun’s study [21], Cronbach’s α = 0.70, and in this study, the tool’s reliability coefficient was Cronbach’s α = 0.86.

#### 2.3.3. Mediating Variable: Self-Stigma

Self-stigma was assessed using the Diabetes Self-Stigma Measurement Tool developed by Seo and Song [22]. This tool comprised 16 items, each measured on a 5-point Likert scale. A higher score indicated higher diabetes self-stigma. However, in the mediation analysis, a reversed score was used such that a higher score indicated lower diabetes self-stigma. At the time of tool development, the tool’s reliability coefficient per Seo and Song’s study [22] was Cronbach’s α = 0.89; in this study, the tool’s reliability coefficient was Cronbach’s α = 0.94.

### 2.4. Data Collection

This study’s data were collected from 1 November to 28 December 2023, targeting patients with type 2 diabetes who visited public health and medical centers in C Province, S City, and D City. Before administering the survey, we met the person in charge of the relevant institution, explained the study’s purpose and procedure, and obtained permission to administer the survey. A research assistant administered the surveys. After being explained the study’s purpose and necessity, the research assistant received training on the questionnaire and then conducted the survey. Participants who were older adults or had poor eyesight were instructed to check their answers after the research assistant read the questionnaire. A small gift was provided to all the participants.

### 2.5. Ethical Consideration

This study’s protocol received approval (JIRB-2023100402-01-231018) from the J University Institutional Review Board. A research consent form was provided on the front page of the questionnaire, and the research consent form stated the survey’s background, purpose, anonymity, and confidentiality and that the survey results would not be used for purposes other than research and would be destroyed upon study completion. Additionally, it was explained that refusing to participate in the study or withdrawing participation during the study would bear no disadvantages. After receiving the completed questionnaire from the research assistant, the researchers directly entered the consent and survey results into an Excel file and stored it in a locked location with a password (to prevent leakage) in a location that only the researchers could access. The research data will be stored for 3 years from the time of study completion, in accordance with Article 15 of the Enforcement Regulations of the Bioethics Act, and then shredded.

### 2.6. Statistical Analysis

The collected data were analyzed using SPSS/WIN 24.0. The participants’ general characteristics and degree of self-stigma, self-efficacy, and coping strategies were analyzed using descriptive statistics. Correlations among self-stigma, self-efficacy, and coping strategies were analyzed using Pearson’s correlation coefficients. Self-stigma’s mediating effect on the relationship between self-efficacy and coping strategies was analyzed using the three-step regression analysis suggested by Baron and Kenny [23]. In Baron and Kenny’s three-step regression analysis method, in the first step, the effect of the independent variable on the mediator was regressed, and in the second step, the effect of the independent variable on the dependent variable was regressed to check for a significant effect. In the third step, the independent variable and mediator were inputted to check their effect on the dependent variable. At this time, the influence of the independent variable was reduced compared to the second step, and the mediator significantly influenced the dependent variable; therefore, a mediating effect was judged. However, if the influence of the independent variable on the dependent variable was significant, it was considered a partial mediation effect; if it was not significant, it was considered a complete mediation effect. The significance of the mediating effect of self-stigma on the relationship between self-efficacy and coping strategies was analyzed using the Sobel test. The significance of the mediation effect was evaluated by bootstrapping using the PROCESS Macro program. The number of bootstrap samples was set to 1000 using a 95% confidence interval. If the confidence interval did not include 0, the mediation effect was considered significant.

## 3. Results

### 3.1. General Characteristics and Degree of Study Variables

This study’s participants were predominantly men (52.9%), and the average age was 67.42 (±12.51) years, with 31.2% being in the 65–74 age group. The highest percentage of participants had graduated from high school (33.3%), followed by elementary school (25.4%). Further, 17.5% lived alone, and 65.1% responded that their perceived economic status was average. The average duration of diabetes was 12.4 (±9.95) years, and the group with less than 10 years was the highest at 50.8%. The most common type of medical institution for treating diabetes was hospitals (61.4%), and only 12.2% of the participants used insulin. Diabetes complications occurred in 14.8% of the patients, and 21.7% had received diabetes education. The mean self-efficacy, coping strategy, and self-stigma scores were 6.29 (±10.80), 2.72 (±0.82), and 2.84 (±0.54), respectively (Table 1).

### 3.2. Difference Between Self-Stigma, Self-Efficacy, and Coping Strategy According to General Characteristics

Self-stigma differed depending on the educational level, perceived economic status, type of hospital, and the presence or absence of complications. Those with a high school diploma or lower had a higher self-stigma than those with a college diploma or higher, and the more dissatisfied they were with their economic status, the higher their self-stigma. Depending on the type of hospital, those receiving treatment at public health centers had the highest self-stigma, whereas those receiving treatment at clinics had the lowest self-stigma. Those with diabetes complications had a higher self-stigma than those without.

Self-efficacy was higher in those who were satisfied with their perceived economic status than in those in the middle and dissatisfied groups. Moreover, those receiving treatment at hospitals and clinics had a higher self-efficacy than those receiving treatment at public health centers.

Coping strategies differed depending on the educational level, perceived economic status, and type of hospital. As with self-stigma, those with a high school diploma or lower had a lower coping strategy degree than those with a college diploma or higher, and those with an average or dissatisfied economic status had a lower coping strategy degree than those who were satisfied. Additionally, those receiving treatment at public health centers had a lower coping strategy degree than those receiving treatment at hospitals or clinics (Table 2).

### 3.3. Correlation Between Self-Stigma, Self-Efficacy, and Coping Strategy

Self-efficacy was negatively correlated with coping strategies (r = −0.52, *p* < 0.001) and self-stigma (r = −0.45, *p* < 0.001): that is, the higher the self-efficacy, the higher the coping strategies, and the lower the self-stigma. Coping strategy was positively correlated with self-stigma (r = 0.78, *p* < 0.001), implying that the higher the coping strategies, the lower the self-stigma (Table 3).

### 3.4. Mediating Effect of Self-Stigma on Relationship Between Self-Efficacy and Coping Strategy

**Figure 1:**
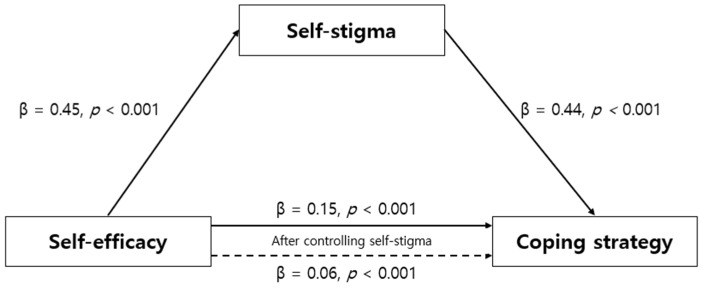
Mediating effect of self-stigma on the relationship between self-efficacy and coping strategy.

To verify the mediating effect of self-stigma on the relationship between self-efficacy and coping strategies of patients with type 2 diabetes in this study, a three-step regression analysis was conducted according to the procedure suggested by Baron and Kenny [23]. Before verifying the mediating effect, the Durbin–Watson value was checked to verify the independence of the residuals; it was 1.65–1.70, which was close to 2, indicating that the dependent variable had no autocorrelation. Furthermore, a multicollinearity assessment revealed that the tolerance limit was 0.79, which was less than 1.0, and the variance inflation factor was 1.25, which was not greater than 10, indicating no multicollinearity concerns and confirming that the model was suitable for regression analysis. The three-step regression analysis results used to verify the mediating effect of self-stigma are presented in Table 4. The regression analysis in step 1 confirmed that the independent variable, self-efficacy, significantly impacted the mediating variable, self-stigma (β = 0.45, *p* < 0.001). In the second-stage regression analysis, the effect of the independent variable, self-efficacy, on the dependent variable, coping strategy, was significant (β = 0.52, *p* < 0.001). In the final stage, to analyze the effect of the mediator, self-stigma, on the dependent variable, coping strategy, a regression analysis was conducted with self-efficacy and self-stigma as the independent variables and coping strategy as the dependent variable. The results revealed that both self-efficacy (β = 0.21, *p* < 0.001) and self-stigma (β = 0.68, *p* < 0.001) impacted the coping strategy. If both the independent variable and mediator were significant in the third stage and the coefficient of the independent variable, self-efficacy, in the third stage was smaller than the coefficient of self-efficacy in the second stage, the mediator could be considered to have a partial mediating effect on the relationship between the independent and dependent variables (Figure 1). That is, self-efficacy had a direct positive effect on coping strategy and also an indirect effect that contributed to improving coping strategies by reducing self-stigma. The explanatory power of this mediating effect was 64.0%. The Sobel test, conducted to verify the significance of the mediating effect of self-stigma, demonstrated self-stigma as a partial mediating variable (Z = 12.51, *p* < 0.001) in the relationship between self-efficacy and coping strategies. Examining the significance of the indirect mediating effect using bootstrapping analysis indicated that the effect size was 0.09, the lower confidence interval was 0.06, and the upper confidence interval was 0.12, which did not include 0, thus suggesting that the effect was significant (Table 5).

## 4. Discussion

This study identified the relationships between self-efficacy, coping strategy, and self-stigma among patients with type 2 diabetes and analyzed the mediating effect of self-stigma. Based on these results, we hereby discuss the effects of self-stigma and self-efficacy on coping strategy.

All three independent variables used herein differed depending on the economic level and type of hospital, and coping strategies and self-stigma differed depending on educational level. Self-efficacy, coping strategies, and self-stigma were higher, better, and lower, respectively, among those with higher economic status, indicating that economic instability affected diabetes management. Previous studies have demonstrated that economic problems impact disease treatment [24,25]. Accordingly, South Korea already provides diabetes patients with the supplies necessary for diabetes management [26]. However, the economic difficulties that they experience are diverse. Therefore, policies reflecting the various economic situations of patients with diabetes should be established. Additionally, the study variables revealed differences in the types of medical facilities where they received treatment. Self-efficacy was the highest among participants receiving treatment at private clinics, coping strategy degree was adequate, and self-stigma was low. By contrast, participants receiving treatment at public health centers had lower self-efficacy, a lower coping strategy degree, and higher self-stigma. This can be considered a difference in doctors’ individualized strategies for the participants. In hospitals, the relationship between the patients and doctors may be stronger. However, in university hospitals, one doctor sees several patients, rendering it difficult to approach them individually. This aspect may also be affected by the economic status, educational level, and disease severity. Patients with lower educational levels have limited access to medical information and resources compared to those with higher education, which may precipitate negative effects on disease and disease management. These results suggest that an individualized approach that considers the diverse backgrounds of patients with diabetes is necessary. Therefore, when contacting patients with diabetes in clinical practice, their personal backgrounds should be considered.

Self-efficacy is a key psychological resource in chronic disease management [27]. Our study found that self-efficacy was significantly related to self-stigma and coping strategies and impacted each variable. This finding suggests that self-efficacy directly affects how patients cope with diabetes-related stress. Previous studies have reported that self-efficacy contributes to reducing self-stigma and coping with stress [28,29]. However, our study indicated that high self-efficacy directly reduces self-stigma and contributes to improving coping strategies for diabetes management. This suggests that, as self-efficacy increases, patients are more likely to view themselves positively, which results in the selection of more effective and proactive coping strategies. Therefore, strategies to enhance self-efficacy should be implemented early on to improve coping among patients with diabetes.

In this study, self-stigma was significantly correlated with (and impacted) coping strategy. That is, when self-stigma is high, the coping strategy degree is low, suggesting that self-stigma acts as a psychological barrier which prevents patients from effectively coping with the stress related to their condition. Specifically, patients with high self-stigma tend to view their illness negatively and have reduced intrinsic motivation for disease management, precipitating the use of ineffective and negative coping strategies (e.g., avoidance and resignation). These results are consistent with previous studies showing that self-stigma among patients with chronic diseases is closely related to depression, stress, and decreased quality of life, all of which negatively affect overall health outcomes [30]. A study on mental health patients reported that self-stigma is related to decreased quality of life and avoidance coping strategies [27]. Therefore, when patients with diabetes use negative coping strategies, their degree of self-stigma must be examined. Additionally, psychological intervention to reduce self-stigma is essential to help patients with type 2 diabetes develop positive coping strategies. Strategies to reduce self-stigma may help patients view their conditions more positively, regain confidence in managing their health, and improve treatment adherence. Interventions to reduce self-stigma in patients with chronic conditions, such as mental illness, have facilitated significant improvements in treatment adherence [31]. Moreover, a study on patients with type 2 diabetes found that higher self-stigma is associated with decreased self-care behaviors [32], suggesting that interventions aimed at reducing self-stigma are essential for improving disease management and health outcomes.

In this study, self-efficacy impacted self-stigma, and self-stigma affected by self-efficacy influenced coping strategy. That is, when self-efficacy is high, self-stigma decreases, resulting in positive coping strategies. Previous research has indicated that patients with high self-efficacy demonstrate higher adherence to blood sugar control, better health behaviors, and the prevention of complications, precipitating positive outcomes in sustainable health management [33]. For instance, intervention programs that enhance self-efficacy over the long term have been proven effective in boosting patients’ confidence and encouraging the consistent practice of health behaviors [34]. By contrast, patients with low self-efficacy tend to avoid or stop necessary health behaviors because of a lack of confidence and trust in their actions [35,36]. These findings align with this study’s results, wherein low self-efficacy was associated with increased self-stigma and weakened coping strategies. This suggests that even in patients with chronic diseases, increased self-efficacy may foster a more positive self-view and the adoption of more effective coping strategies, consistent with previous studies [36,37]. Therefore, self-efficacy and self-stigma play important roles in the formation of positive coping strategies for sustainable chronic disease management. Therefore, implementing a program that reflects the elements of self-stigma in intervention programs to improve self-efficacy among patients with type 2 diabetes is an important approach to improving disease management success.

## 5. Conclusions

This study’s findings demonstrate that self-stigma negatively partially mediates the relationship between self-efficacy and coping strategies in individuals with type 2 diabetes. Thus, it contributes to the understanding of the relationship between self-efficacy, coping strategy, and self-stigma and offers important intervention strategies to improve coping among patients with diabetes. However, as this study is cross-sectional, it has limitations in fully understanding the temporal relationships between these variables. Therefore, we recommend that longitudinal studies be conducted to better understand the interactions between self-efficacy, coping strategies, and self-stigma and examine the long-term effects of these variables. Longitudinal studies are needed to understand how self-stigma reduction and improved self-efficacy affect disease management over time and develop more effective interventions for type 2 diabetes.

## Figures and Tables

**Table 1:** Participants’ general characteristics and study variables (*n* = 189).

Characteristics	Categories	M ± SD or *n* (%)	Min–Max
Gender	Male	100 (52.9)	
	Female	89 (47.1)	
Age (yr)		67.42 ± 12.51	32.00–94.00
	<55	31 (14.4)	
	55~64	42 (22.2)	
	65~74	59 (31.2)	
	≥75	57 (30.2)	
Educational level	Elementary school or lower	48 (25.4)	
	Middle school	37 (19.6)	
	High school	63 (33.3)	
	≥University or higher	41 (21.7)	
Living type	Alone	33 (17.5)	
	With family	156 (82.5)	
Perceived economic status	Satisfied	35 (18.5)	
	Moderate	123 (65.1)	
	Unsatisfied	31 (16.4)	
Duration of diabetes (yrs)		12.40 ± 9.95	1.00–43.00
	≤10	96 (50.8)	
	11~20	53 (28.0)	
	≥21	40 (21.2)	
Type of hospital used for treatment	Public health center	45 (23.8)	
	Hospital	116 (61.4)	
	Clinic	28 (14.8)	
Type of medication	PO	166 (87.8)	
	Insulin	7 (3.7)	
	PO + Insulin	16 (8.5)	
Having complications	Yes	28 (14.8)	
	No	161 (85.2)	
Having received education on diabetes	Yes	148 (78.3)	
	No	41 (21.7)	
Self-efficacy		6.29 ± 10.80	1.88–10.00
Coping strategy		2.72 ± 0.82	1.00–5.00
Self-stigma		2.84 ± 0.54	1.38–4.14

M = mean; SD = standard deviation; Min = minimum; Max = maximum; and PO = per os.

**Table 2:** Differences in diabetes self-efficacy, coping strategy, and self-stigma according to general characteristics (*n* = 189).

Characteristics	Categories	Self-Efficacy			Coping Strategy			Self-Stigma		
		M ± SD	t or F	*p* Scheffe	M ± SD	t or F	*p* Scheffe	M ± SD	t or F	*p* Scheffe
Gender	Male	6.26 ± 1.95	−0.24	0.812	2.83 ± 0.55	−0.45	0.656	2.65 ± 0.80	−1.27	0.207
	Female	6.32 ± 1.61			2.86 ± 0.53			2.80 ± 0.85		
Age (yr)	<55 ^a^	6.03 ± 1.70	1.08	0.358	2.77 ± 0.57	1.96	0.122	2.57 ± 0.86	2.48	0.063
	55~64 ^b^	6.45 ± 1.78			2.71 ± 0.56			2.48 ± 0.89		
	65~74 ^c^	6.55 ± 1.76			2.95 ± 0.52			2.85 ± 0.75		
	≥75 ^d^	6.05 ± 1.88			2.87 ± 0.52			2.84 ± 0.79		
Educational level	Elementary school ^a^	5.94 ± 1.80	2.45	0.065	2.94 ± 0.42	5.14	0.002 a, b, c > d	2.88 ± 0.69	7.78	<0.001 a, b, c > d
	Middle school ^b^	6.16 ± 1.87			2.94 ± 0.52			2.96 ± 0.78		
	High school ^c^	6.22 ± 1.74			2.89 ± 0.58			2.80 ± 0.83		
	≥University ^d^	6.92 ± 1.71			2.56 ± 0.54			2.21 ± 0.82		
Living type	Yes	6.01 ± 1.84	0.94	0.352	2.90 ± 0.59	0.62	0.539	2.81 ± 0.89	0.65	0.521
	No	6.35 ± 1.79			2.83 ± 0.53			2.70 ± 0.81		
Perceived Economic status	Satisfied ^a^	7.36 ± 1.62	11.17	<0.001 a > b,c	2.47 ± 0.60	13.51	<0.001 a < b, c	2.13 ± 0.73	14.47	<0.001 a < b < c
	Moderate ^b^	6.21 ± 7.78			2.88 ± 0.50			2.80 ± 0.81		
	Unsatisfied ^c^	5.41 ± 1.46			3.10 ± 0.42			3.08 ± 0.62		
Duration of diabetes (yr)	≤10 ^a^	6.41 ± 1.67	0.43	0.648	2.82 ± 0.56	0.45	0.638	2.68 ± 0.83	1.73	0.181
	11~20 ^b^	6.22 ± 1.96			2.90 ± 0.53			2.63 ± 0.80		
	≥21 ^c^	6.11 ± 1.90			2.81 ± 0.52			2.93 ± 0.82		
Type of hospital	Public health center ^a^	5.37 ± 1.53	10.46	<0.001 a < b, c	3.18 ± 0.43	16.27	<0.001 a > b,c	3.16 ± 0.56	15.44	<0.001 a > b > c
	Hospital ^b^	6.44 ± 1.77			2.78 ± 0.51			2.69 ± 0.83		
	Clinic ^c^	7.15 ± 1.72			2.54 ± 0.55			2.14 ± 0.78		
Type of medication	PO ^a^	6.27 ± 1.81	0.15	0.861	2.84 ± 0.53	1.41	0.247	2.68 ± 0.79	2.03	0.134
	Insulin ^b^	6.52 ± 1.98			3.13 ± 0.59			3.23 ± 0.92		
	PO + insulin ^c^	6.47 ± 1.62			2.73 ± 0.59			2.92 ± 1.02		
Having a complications	Yes	5.97 ± 1.66	1.09	0.285	2.99 ± 0.51	−1.61	0.115	3.12 ± 0.81	−2.81	0.008
	No	6.35 ± 1.82			2.82 ± 0.54			2.65 ± 0.81		
Experience of diabetes education	Yes	6.32 ± 1.78	−0.46	0.648	2.86 ± 0.56	−0.94	0.350	2.78 ± 0.86	−1.70	0.091
	No	6.17 ± 1.88			2.78 ± 0.46			2.53 ± 0.67		

**Table 3:** Correlations among self-stigma, self-efficacy, and coping strategy (*n* = 189).

	1 r (*p*)	2 r (*p*)	3 r (*p*)
1. Self-efficacy	1		
2. Coping strategy	−0.52 (<0.001)		
3. Self-stigma	−0.45 (<0.001)	0.78 (<0.001)	

**Table 4:** Mediating effect of self-stigma on the relationship between self-efficacy and coping strategy (*n* = 189).

Step	Independent Variables	Dependent Variables	B	SE	β	t (*p*)	Adj. R^2^	F (*p*)
1	Self-efficacy	Self-stigma	0.21	0.03	0.45	6.95 (<0.001)	0.201	45.34 (<0.001)
2	Self-efficacy	Coping strategy	0.15	0.01	0.52	17.73 (<0.001)	0.268	69.74 (<0.001)
3	Self-efficacy	Coping strategy	0.06	0.01	0.21	4.29 (<0.001)	0.640	167.92 (<0.001)
	Self-stigma	Coping strategy	0.44	0.03	0.68	13.93 (<0.001)		
			Sobel test; Z = 12.51 (*p* < 0.001)					

Adj. = adjusted; SE = standard error.

**Table 5:** Statistical significance of indirect mediation effects (*n* = 189).

Effect	Boot SE	95% Confidence Interval	
		Boot LLCI	Boot ULCI
0.09	0.02	0.06	0.12

SE = standard error; LLCI = lower confidence interval; and ULCI = upper-level confidence interval.

## Data Availability

The original contributions presented in this study are included in the article. Further inquiries can be directed to the corresponding author(s).

## References

[1] International Diabetes Federation, IDF. https://idf.org/about-diabetes/diabetes-facts-figures/.

[2] Korean Diabetes Association (2020). Diabetes Fact Sheet in Korea 2024.

[3] Korea Disease Control and Prevention Agency, KDCA. https://health.kdca.go.kr/healthinfo/biz/health/gnrlzHealthInfo/gnrlzHealthInfo/gnrlzHealthInfoView.do?cnnts_sn=2351.

[4] Papatheodorou K., Banach M., Bekiari E., Rizzo M., Edmonds M. (2018). Complications of diabetes 2017. J. Diabetes Res..

[5] Singh P., Khullar S., Singh M., Kaur G., Mastana S. (2015). Diabetes to cardiovascular disease: Is depression the potential missing link?. Med. Hypotheses.

[6] Bandura A. (1986). The explanatory and predictive scope of self-efficacy theory. J. Soc. Clin. Psychol..

[7] Al-Amer R., Ramjan L., Glew P., Randall S., Salamonson Y. (2016). Self-efficacy, depression, and self-care activities in adult Jordanians with type 2 diabetes: The role of illness perception. Issues Ment. Health Nurs..

[8] Sheeran P., Maki A., Montanaro E., Avishai-Yitshak A., Bryan A., Klein W.M., Miles E., Rothman A.J. (2016). The impact of changing attitudes, norms, and self-efficacy on health-related intentions and behavior: A meta-analysis. Health Psychol..

[9] Sukarno A., Bahtiar B. (2022). The effectiveness of cognitive behavior therapy on psychological stress, physical health, and self-care behavior among diabetes patients: A systematic review. Health Educ. Health Promot..

[10] Zaheri H., Najar S., Abbaspoor Z. (2017). Effectiveness of cognitive-behavioral stress management on psychological stress and glycemic control in gestational diabetes: A randomized controlled trial. J. Matern. -Fetal Neonatal Med..

[11] McCoy M.A., Theeke L.A. (2019). A systematic review of the relationships among psychosocial factors and coping in adults with type 2 diabetes mellitus. Int. J. Nurs Sci..

[12] Hapunda G. (2022). Coping strategies and their association with diabetes specific distress, depression and diabetes self-care among people living with diabetes in Zambia. BMC Endocr. Disord..

[13] Schoenmakers E.C., Van Tilburg T.G., Fokkema T. (2015). Problem-focused and emotion-focused coping options and loneliness: How are they related?. Eur. J. Ageing..

[14] Eisenberg M.H., Lipsky L.M., Dempster K.W., Liu A., Nansel T.R. (2016). I should but I can’t: Controlled motivation and self-efficacy are related to disordered eating behaviors in adolescents with type 1 diabetes. J. Adolesc. Health.

[15] Novitasari E., Hamid A.Y.S. (2021). The relationships between body image, self-efficacy, and coping strategy among Indonesian adolescents who experienced body shaming. Enfermería Clínica..

[16] Seo K., Song Y. (2019). Self-stigma among Korean patients with diabetes: A concept analysis. J. Clin. Nurs..

[17] Han E., Scior K., Avramides K., Crane L. (2022). A systematic review on autistic people’s experiences of stigma and coping strategies. Autism Res..

[18] Lorig K., Stewart A., Ritter P., Gonzalez V., Laurent D., Lynch J. (1996). Outcome Measures for Health Education and Other Health Care Interventions.

[19] Chang S.J., Song M., Im E.O. (2014). Psychometric evaluation of the Korean version of the Diabetes Self-efficacy S ale among South Korean older adults with type 2 diabetes. J. Clin. Nurs..

[20] Welch G. (1994). The Diabetes Coping Measure: A measure of cognitive and behavioral coping specific to diabetes. Handbook Psychology and Diabetes: A Guide to Psychological Measurement in Diabetes Research and Practice.

[21] Byun S.H. (2015). Structural Equation Modeling for Quality of Life with Diabetes: Associated with Diabetes Locus of Control, Social Support, Self-Efficacy, and Coping Strategy. Unpublished. Ph.D. Thesis.

[22] Seo K., Song Y. (2021). Development and validation of the self-stigma scale in people with diabetes. Nurs. Open..

[23] Baron R.M., Kenny D.A. (1986). The moderator-mediator variable distinction in social psychological research: Conceptual, strategic, and statistical considerations. J. Pers. Soc. Psychol..

[24] Banu B., Khan M.M.H., Ali L., Barnighausen T., Sauerborn R., Souares A. (2024). Pattern and predictors of non-adherence to diabetes self-management recommendations among patients in peripheral district of Bangladesh. Trop. Med. Int. Health.

[25] Ha J.H., Jin H., Park J.U. (2021). Association between socioeconomic position and diabetic foot ulcer outcomes: A population-based cohort study in South Korea. BMC Public. Health.

[26] Yoo J.H. (2024). 2024 Policy Revision for Support of Type 1 Diabetes Mellitus Patients. J. Korean Diabetes.

[27] Liu F., Deng H., Hu N., Huang W., Wang H., Liu L., Chai J., Li Y. (2024). The relationship between self-stigma and quality of life in long-term hospitalized patients with schizophrenia: A cross-sectional study. Front. Psychiatry.

[28] Aktu Y., Aras E. (2024). Adaptation and validation of the Parents’ Self-stigma Scale into Turkish and its association with parenting stress and parental self-efficacy. BMC Psychol..

[29] Bakan G., Inci F.H. (2021). Predictor of self-efficacy in individuals with chronic disease: Stress-coping strategies. J. Clin. Nurs..

[30] Wang R.H., Lin C.C., Chen S.Y., Hsu H.C., Huang C.L. (2021). The impact of self-stigma, role strain, and diabetes distress on quality of life and glycemic control in women with diabetes: A 6-month prospective study. Biol Res Nurs..

[31] Kim J., Na H. (2016). The relationship between internalized stigma and treatment adherence in community-dwelling individuals with mental illness: The mediating effect of self-efficacy. J. Psychiatr. Nurs..

[32] Kato A., Fujimaki Y., Fujimori S., Isogawa A., Onishi Y., Suzuki R., Yamauchi T., Ueki K., Kadowaki T., Hashimoto H. (2016). Association between self-stigma and self-care behaviors in patients with type 2 diabetes: A cross-sectional study. BMJ Open Diabetes Res. Care..

[33] Keum H., Suh S., Han S. (2020). The Influence of Self-management Knowledge and Distress on Diabetes Management Self-efficacy in Type 2 Diabetes Patients. J. Korea Acad. -Ind. Coop. Soc..

[34] Ataya J., Soqia J., Albani N., Tahhan N.K., Alfawal M., Elmolla O., Albaldi A., Alsheikh R.A., Kabalan Y. (2024). The role of self-efficacy in managing type 2 diabetes and emotional well-being: A cross-sectional study. BMC Public Health.

[35] Babazadeh T., Lotfi Y., Ranjbaran S. (2023). Predictors of self-care behaviors and glycemic control among patients with type 2 diabetes mellitus. Front. Public. Health.

[36] Ong-Artborirak P., Seangpraw K., Boonyathee S., Auttama N., Winaiprasert P. (2023). Health literacy, self-efficacy, self-care behaviors, and glycemic control among older adults with type 2 diabetes mellitus: A cross-sectional study in Thai communities. BMC Geriatr..

[37] Xing S., Liu Y., Zhang H., Li B., Jiang X. (2023). The mediating role of diabetes stigma and self-efficacy in relieving diabetes distress among patients with type 2 diabetes mellitus: A multicenter cross-sectional study. Front. Psychol..

